# Smooth muscle contractility of laser-enucleated prostate tissues and impacts of preoperative α_1_-blocker treatment in patients with and without catheterization

**DOI:** 10.1038/s41598-025-88884-7

**Published:** 2025-02-10

**Authors:** Patrick Keller, Sheng Hu, Laurenz Berger, Philip Nicola, Felix Schierholz, Alexander Tamalunas, Oluwafemi E. Kale, Philipp Weinhold, Raphaela Waidelich, Christian G. Stief, Martin Hennenberg

**Affiliations:** 1 Department of Urology, LMU University Hospital, LMU Munich, Munich, Germany; 2 Urologische Klinik Und Poliklinik, Marchioninistr. 15, 81377 München, Germany

**Keywords:** Benign prostatic hyperplasia (BPH), Prostate smooth muscle, Lower urinary tract symptoms (LUTS), Laser enucleation of the prostate, α_1_-blocker, Pharmacology, Prostate, Benign prostatic hyperplasia, Urinary tract obstruction

## Abstract

Prostate smooth muscle contraction is central in treatment of voiding symptoms in benign prostatic hyperplasia (BPH). Tissues from transurethral resection of the prostate (TURP) and radical prostatectomy (RP) for prostate cancer are widely used to study contractions. However, findings are limited by traumatization in TURP, and uncertain relationship to BPH in RP tissues. This study aims to examine contractions of laser-enucleated tissues. Tissues from holmium/thulium laser enucleation (HoLEP/ThuLEP) and TURP were contracted by KCl, noradrenaline and electric field stimulation (EFS) in an organ bath. Contractions were compared to RP tissues in previous studies. KCl-induced contractions averaged 2.5 mN, 0.7 mN and 3.3 mN in tissues from HoLEP/ThuLEP, TURP and RP, with non-responsive tissues included (2.4% HoLEP/ThuLEP, 37% TURP). Maximum EFS-induced contractions (E_max_) averaged 47% of KCl in HoLEP/ThuLEP tissues, 27% in TURP tissues, and 68–235% in 21 previous studies with RP tissues. E_max_ values for noradrenaline averaged 99.7% in HoLEP/ThuLEP tissues, 56% in TURP tissues, and ranged from 92 to 260% in RP tissues. Preoperative α_1_-blocker treatment reduced EFS- and noradrenaline-induced contractions, and increased EC_50_ values for noradrenaline in laser-enucleated, catheterized patients, but not in patients without catheterization. Also, the ex vivo application of α_1_-blockers increased the EC_50_ values for noradrenaline and reduced E_max_ for EFS. Laser-enucleated tissues allow investigation of prostate smooth muscle contraction in medication-refractory voiding symptoms. Different impacts of preoperative α_1_-blocker treatment on ex vivo contractility in tissues from patients with and without catheterization point to clinically relevant heterogeneity of patients undergoing surgery for BPH.

## Introduction

Prostate smooth muscle contraction is considered essential in the pathophysiology and medical treatment of voiding symptoms suggestive of benign prostatic hyperplasia (BPH)^1,2^. Increased prostate smooth muscle tone may contribute to urethral obstruction, resulting in impaired bladder emptying, and finally in symptoms^1,2^. The α_1_-adrenoceptor antagonists (α_1_-blockers), applied for rapid symptom improvement, represent the first line option for medical treatment and are believed to act by inhibition of α_1_-adrenergic prostate smooth muscle contraction^2,3^. The phosphodiesterase-5 inhibitor tadalafil is available as an alternative and is believed to improve symptoms by smooth muscle relaxation as well^3^. Treatment with 5α-reductase inhibitors (5ARI) is recommended for prevention of progression, complications and surgery in BPH^3^.

However, improvements by available drugs underlay obvious ceilings. The α_1_-blockers reduce symptom scores by maximally 30–50% and enhance the maximum urinary flow rate (Q_max_) by not more than 40%^2,3^. These improvements are not far from placebos, reducing symptom scores by 10–34%, and increasing the Q_max_ up to 28%^2,3^. Tadalafil decreases symptom scores to similar extent as α_1_-blockers, but does not enhance Q_max_ in most studies^3^. During prolonged application 5ARIs may improve symptoms as well, but benefits are hardly or not additive with α_1_-blockers. Low adherence, attributed to insufficient efficacy and unbalanced side effects accounts for the progression and complications, hospitalization, and finally contributes to high numbers of surgery due to BPH^2,3^. Surgery often becomes inevitable with progression of BPH and despite drug treatment, in patients with imminent complications but also as last resort treatment for adequate symptom improvement^3,4^. Transurethral resection of the prostate (TURP) was the standard surgery for BPH for decades, while holmium and thulium laser enucleation of the prostate (HoLEP, ThuLEP) are increasingly established alternatives^3,4^.

Limited drug efficacy in voiding symptoms suggestive of BPH raised ongoing preclinical research addressing prostate smooth muscle contraction. Currently available drugs and drug candidates were developed based on experimental studies investigating their effects on contractions of prostate tissues in vitro, including human tissues. Tissue models included human tissues from radical prostatectomy (RP) for prostate cancer (PCa), or from TURP. However, TURP tissues have been supposed to be traumatized, by heat-induced denaturation during surgery, while RP tissues are not specifically representative for BPH, and do not cover medication-refractory voiding symptoms in BPH. To assess the potential of samples from laser enucleation for preclinical investigations, this study aims to examine the contractility of prostate tissues from HoLEP and ThuLEP.

## Materials and methods

### Study design, strategy and aims

This study was carried out in accordance with the Declaration of Helsinki of the World Medical Association and has been approved by the ethics committee of the Ludwig-Maximilians University, Munich, Germany (approval number 22-0608, from 08-10-2022). Informed consent was obtained from all patients. Samples and data were collected and analyzed pseudonymized. This study included two parts. Firstly, tissues were collected from laser enucleation (HoLEP, ThuLEP) and TURP, and randomly assigned to examination of electric field stimulation- (EFS)- and noradrenaline-induced contractions. The primary aims of this part were to explore whether tissues from laser nucleation are still contractile in organ bath experiments, and a comparison to TURP and RP tissues. Experiments with TURP tissues were discontinued after experiments with tissues from 43 patients, as it became obvious that a high percentage did not react to KCl. Experiments with laser-enucleated tissues were continued until tissues from 49 patients were examined with EFS, 59 patients with noradrenaline, and 2 patients with KCl but without showing contractions, all obtained from a total of 85 laser-enucleated patients. Laser-enucleated and TURP samples without reaction to KCl were included in frequency and concentration response curves and for calculation of E_max_ values, i. e. contractions in these samples were rated as 0 mN at each frequency and each noradrenaline concentration. KCl-induced contractions in samples from laser enucleation and TURP were compared to values from RP tissues in two of our previous study results^5,6^. Further, the E_max_ values for EFS and noradrenaline in samples from laser enucleation were compared to E_max_ values from our previous study results with RP tissues published 2018–2023^5–25^. EFS- and noradrenaline-induced contractions in laser-enucleated tissues were analyzed for different subgroups, including separation for patients with and without catheterization for urinary retention, and preoperative treatment with α_1_-blockers, after completion of all experiments.

Secondly, tissues from laser enucleation were collected from 21 further patients (not participating in the first part), and examined with EFS or noradrenaline, after the addition of α_1_-blockers or solvent (controls) in the organ bath. Thus, these experiments were planned and implemented after completion of the first part, as it turned out that laser-enucleated tissues are contractile in the organ bath. This second part was to assess, whether these tissues from laser enucleation are still suitable to examine effects of anticontractile drug candidates.

### Holmium and thulium laser enucleation of the prostate

HoLEP enucleation was performed in a three-lobe technique, using the VersaPulse^®^ 100W Holmium Laser (Lumenis Ltd., Yokneam, Israel) with a frequency of 53 Hz and a power setting of 1.2 kJ, or the 150 W CyberHo Holmium laser paired with a 550 micron end-firing laser fiber, utilizing energy settings of 2.0 J per puls and a frequency of 50 Hz. For ThuLEP enucleation, a Dornier Thulio^®^ p-Tm:YAG with 570 micron end-firing disposable fiber was used with energy settings of 1.5 J per puls and a frequency of 50 Hz, resulting in 75 W. Tissue morcellation was performed using dual 5 mm reciprocating hollow metal blades. The same three-lobe technique was used for both the holmium and thulium laser enucleation^26^. Enucleation of the median lobe started distally and progressed proximally towards the bladder neck. Once detached, the median lobe was pushed into the bladder, and prostatic attachments were released from the bladder neck, allowing the median lobe to fall into the bladder. Enucleation of the lateral lobes proceeded similarly, beginning with the right lateral lobe at the level of the verumontanum. At the 5 o’clock position of the prostate apex, the lateral lobe was detached from the surgical capsule, and pushed into the urinary bladder. Dissection of the left lateral lobe followed a similar protocol, starting at the 7 o’clock position. Identical to the other side, the side flap on the surgical capsule was released using the laser and pushed into the bladder. Tissue morcellation was initiated by the insertion of an offset nephroscope fitted with a soft-tissue morcellator into the bladder, under adequate bladder filling. Tissues were shredded on the circular knife, effectively cutting the adenoma into smaller pieces, which were subsequently suctioned out of the bladder and collected.

### Transurethral resection of the prostate

TURP was performed by bipolar resection. Compared to monopolar systems this leads to a lower resection temperature, and consequently to lower thermal damage in the surrounding tissue. Following the insertion of the resectoscope through the urethra, a high-frequency electrical current was used to remove overgrown tissue of the prostate, layer by layer until reaching the surgical capsule. After excess tissue was removed using a glass syringe, blood vessels were sealed to stop bleeding, and the resectoscope was removed.

### Data from tissues from radical prostatectomy

For comparison of tissues from TURP and laser enucleation with tissues from RP, E_max_ values for EFS- and noradrenaline-induced contractions were compiled from control groups in previous studies with RP tissues from our lab (2018–2023)^5–25^. E_max_ values were collected from each single experiment, with each value representing the mean of two samples from the same prostate, or the value of a single determination if only one sample was available in an independent experiment. Values were obtained from the control groups of these previous studies and were consequently obtained with solvents (mostly dimethylsulfoxid, DMSO) in varying amounts. Tissues were collected from periurethral zones, as previously described^5,6^. Conditions for interim storage and transport were similar to conditions for tissues from surgery for BPH in this study, with the exception that tissues from RP were macroscopically inspected and sampled by pathologists.

### Organ bath experiments

**Fig. 1:**
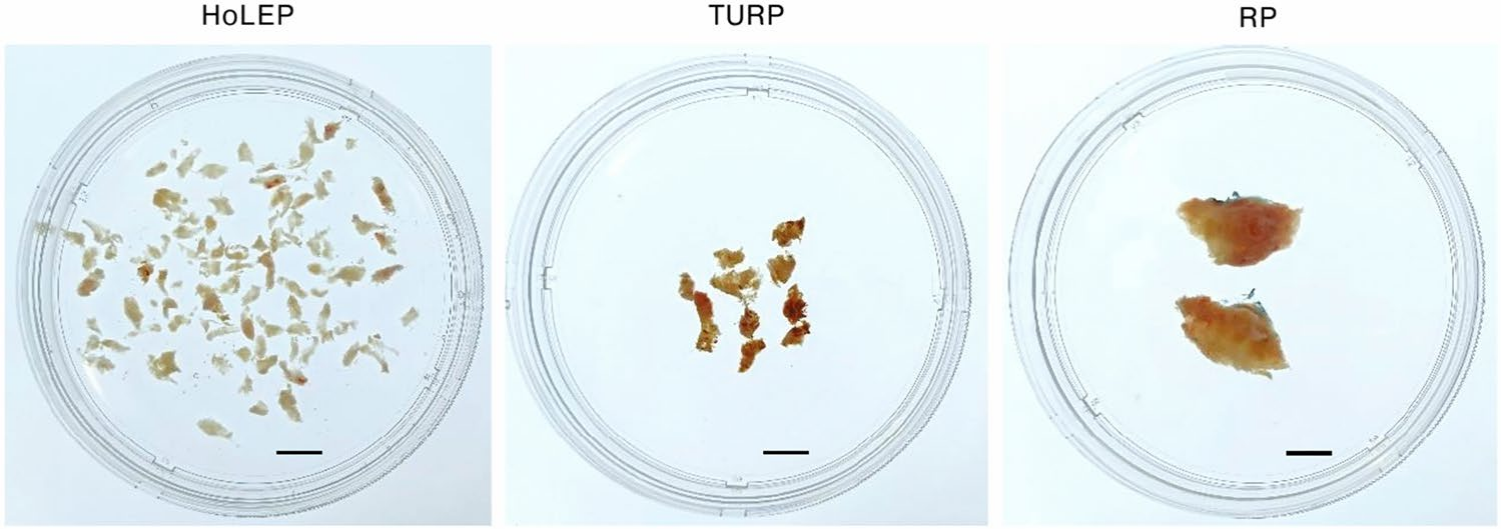
Tissues collected from HoLEP, TURP and RP. Content of one dish represents the complete sample being collected from one patient. Scale bars are 1 cm.

Collected tissues varied qualitatively between surgeries (Fig. 1). Macerations from laser-enucleation consisted of numerous small tissue shreds (dozens to hundreds per collected sample), with the largest measuring around 0.5–10 × 4 mm in size (Fig. 1). Tissue shreds matching the required size for organ bath experiments (approximately 6 × 3 × 3 mm) were either used directly without further cutting or prepared by cutting largest available shreds. TURP chips were typically larger than shreds from laser-enucleation, with most chips reaching ≥ 1 cm in length, but often including flat or narrow protrusions (Fig. 1). Strips for experiments were prepared from the largest available chips, specifically from interior regions of chunk-like parts and excluding margins (presumed to be most traumatized), extensions and flattened areas.

Organ bath experiments were performed as recently described for RP tissues^5,6^. Tissue strips were mounted in organ baths, with four chambers per device (model 720 M, Danish Myotechnology, Aahus, Denmark) containing 10 ml Krebs–Henseleit solution (37 °C, pH 7.4) continuously gassed with carbogen (95% O_2_ and 5% CO_2_). After adjustment of a stable pretension of 4.9 mN within 45 min^5,6^, tissues were contracted by 80 mM KCl, by the addition of a 2 M KCl solution. As soon as a maximum plateau contraction was obtained, high molar KCl was washed out, resulting in a new baseline. Subsequently, tissues were used directly for frequency response curves by EFS or concentration response curves for noradrenaline. In the second study part, α_1_-blockers or solvent (for controls) were added after washout of KCl, and frequency or concentration response curves were constructed 30 min later. With each strip, only one frequency or concentration response curve was recorded.

For construction of frequency and concentration response curves (without α_1_-blockers or solvent), strips were intuitively allocated to EFS and noradrenaline. Channels showing no reaction to KCl were not further examined by EFS or with noradrenaline, and included to analyses by rating as 0 mN at each frequency and noradrenaline concentration, except of a separate analysis as indicated. From a total of laser-enucleated tissues from 85 patients, 49 were examined by EFS and 59 with noradrenaline. In tissues from two patients, none of the strips contracted with KCl, which were included in data analyses by rating EFS- and noradrenaline-induced contractions as zero, resulting in tissues from 51 patients analyzed for EFS-induced, and from 61 patients analyzed for noradrenaline-induced contractions. Tissues from most patients were assessed by double or multiple determinations. In 46 out of 51 EFS experiments, 2–4 strips were examined per patient, and the remaining five as single determination with only one strip. In 53 out of 61 noradrenaline experiments, again multiple strips were examined per patient (2–4 strips for tissues from 49 patients, 5–8 strips for tissues from 4 patients). From a total of TURP tissues from 43 patients, 7 were examined by EFS and 26 with noradrenaline. In tissues from 16 patients, none of the examined strips contracted with KCl, which were rated as zero for analyses of EFS- and noradrenaline-induced contractions, resulting in TURP tissues from 23 patients analyzed for EFS, and from 42 patients analyzed for noradrenaline. In 21 out of 23 EFS experiments, multiple strips were examined per patient (2–4 strips for tissues from 18 patients, 6–8 strips for tissues from 3 patients). In 41 out of 42 noradrenaline experiments, multiple strips were examined per patient (2–4 strips for tissues from 28 patients, 5–8 strips for tissues from 13 patients).

Ex vivo effects of α_1_-blockers were assessed in paired samples, i. e. α_1_-blockers or solvent were added to tissue strips from the same patient, being examined in the same experiment. Double determinations for the solvent and antagonist group were possible in 30 out of a total of 41 experiments. In the remaining experiments, the amount of available tissues did not allow the filling of two channels for both groups or single samples did not contract with KCl. However, these experiments included three samples per experiment, split to the control and antagonist group.

Agonist- and EFS-induced contractions are expressed as percentage of 80 mM KCl-induced contractions, as this may correct individual variations and heterogeneities, and variables such as strip size or smooth muscle content. E_max_ values, EC_50_ values for agonists, and frequencies inducing 50% of the maximum EFS-induced contraction (EF_50_) were calculated separately for each single experiment by curve fitting^27^, using GraphPad Prism 6 (GraphPad Software Inc., San Diego, CA, USA). The software sends error messages, if curve fitting is not possible, or if results from curve fitting are suspected as “ambiguous”. In addition, values from curve fitting were checked manually for plausibility, as recommended in the “GraphPad Curve Fitting Guide” (GraphPad Software Inc.). Results in the first study part were marked as “ambiguous” in one EFS and one noradrenaline experiment. Following manual inspection, these values were considered plausible. As curve fitting was not possible with samples without contractions, E_max_ for these tissues was set to 0 mN. Concentration response curves addressing ex vivo effects of α_1_-blockers included noradrenaline concentrations up to 3 mM, to allow detection of rightshifts and recovery at high agonist concentrations, and to allow curve fitting in antagonist groups. Consequently, control curves frequently included downhill parts at high agonist concentrations, which had to be excluded in 8 out of 21 experiments with noradrenaline, to allow plausible curve fitting. Values from 2 curves with tamsulosin, one with noradrenaline and one with EFS, were labelled as “ambiguous”, but provided plausible values after the exclusion of untypical values. Data for catheterization were available from 81 and for premedication from 82 out of 85 patients. Contraction data from patients with unavailable clinical data were excluded from group analyses.

### Statistical analyses

Data in frequency and concentration response curves are means with standard deviation (SD). Single values in scatter plots are either means from all strips examined per tissue, or are values from each single strip. Group differences and effect sizes in the text are reported as mean differences (MD) with 95% confidence intervals (95% CI). Calculation of MDs and 95% CIs, and statistical analyses were performed using GraphPad Prism 6. Distribution of values within data sets including KCl-induced contractions, E_max_, EC_50_ and EF_50_ values were assessed by the D’Agostino & Pearson omnibus normality test (alpha = 0.05). Groups showing Gaussian distribution were compared using parametric tests, while non-parametric tests were applied to data sets containing at least one group not passing the normality test. Comparisons of KCl-induced contractions between three groups, and comparisons of previously reported E_max_ values to E_max_ values in the current study were performed by Dunn’s multiple comparison after one-way ANOVA with Kruskal Wallis test, allowing comparison of multiple groups without normal distribution. E_max_, EC_50_ and EF_50_ values in grouping analyses (i. e., between two groups) were compared by unpaired, two-tailed Mann Whitney test if data were not normally distributed in at least one of both groups, and by unpaired, two-tailed t test if data were normally distributed in both groups. E_max_, EC_50_ and EF_50_ values between paired groups (i. e., with/without ex vivo application of tamsulosin or silodosin) were compared by paired, two-tailed Wilcoxon matched-pairs signed rank test if data were not normally distributed in at least one of both groups, and by paired, two-tailed t test if data were normally distributed in both groups. Comparison of whole frequency and concentration response curves was performed by two-way analysis of variance (ANOVA), without multiple comparison and as data sets included three variables (concentration, contraction, treatment)^27^. *P* values < 0.05 were considered significant. *P* values ≥ 0.05 are not indicated. The present study and analyses show an exploratory design, as typical features of a hypothesis-testing study are lacking, including a clear preset study plan, blinding, or biometric calculation of group sizes^28^. Consequently, *P* values reported here are descriptive, but not hypothesis-testing^28^. While the formation of group sizes was not driven by power calculations, 10 independent experiments per series were consistently found sufficient to detect biologically relevant differences or to detect drug effects in our previous organ bath experiments. Consequently, and as splitting into up to 4 subgroups was intended, while it was anticipated that clinical data would not be available from some patients, experiments with tissues from 50 to 60 patients were aimed with noradrenaline and EFS in part one, and from at least 10 patients per series in part two, without interim analyses.

## Results

### Potassium-induced contractions

**Fig. 2:**
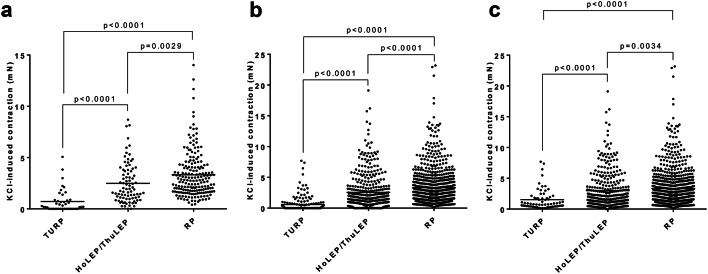
Potassium-induced contractions in tissues from surgery for BPH, and from radical prostatectomy for PCa. Shown are means from each tissue (**a**) (i.e., values represent means, mostly based on investigation of two or more samples from one tissue), values from each examined tissue sample (**b**) (i.e., without calculating means for each prostate tissue), and again values from each examined single sample, but with samples showing no reaction to KCl (i. e., 0 mN) being omitted (**c**). Contractions by 80 mM potassium were induced before proceeding with construction of frequency response curves for EFS, or with concentration response curves for noradrenaline, using tissues from laser enucleation (HoLEP/ThuLEP) or TURP for BPH, or from RP for PCa. Data from RP tissues are from two of our previous studies^5,6^. Data are shown together with means for each groups (bars), and with *P* values from Dunn’s multiple comparison test after one-way ANOVA with Kruskal Wallis test.

Potassium-induced contractions of laser-enucleated tissues from HoLEP and ThuLEP (n = 85 patients) were higher compared to tissues from TURP (n = 43 patients) (HoLEP/ThuLEP 2.49 mN [2.07–2.92]; TURP 0.72 mN [0.38–1.06]; MD 1.77 mN [0.76–2.78]) (Fig. 2a). Potassium-induced contractions of RP tissues (RP 3.32 mN [3–3.64]; n = 189 patients) were higher compared to HoLEP/ThuLEP tissues (MD 0.83 mN [0.13–1.54]), and compared to TURP tissues (MD 2.6 mN [1.69–3.52]) (Fig. 2a). Included to these analyses are tissues and samples showing no reaction to KCl. In 2 out of 85 tissues from laser enucleation (2.4%), none of the examined single samples responded to KCl, while 16 out of 43 tissues from TURP (37.2%) were completely unresponsive to KCl with each examined sample.

These differences persisted, if contractions of all single samples were analyzed instead of means of each prostate. Again, the difference between laser-enucleated and RP tissues was smaller (HoLEP/ThuLEP 2.57 mN [2.27–2.87]; RP 3.33 mN [3.1–3.55]; MD 0.76 mN [0.29–1.26]), than the difference between TURP tissues and both other groups (TURP 0.58 mN [0.38–0.77]; MD 2.75 mN [2.12–3.38] vs. RP; MD 1.99 mN [1.37–2.61] vs. HoLEP/ThuLEP) (Fig. 2b). If samples from HoLEP/ThuLEP tissues showing no reaction to KCl (i. e., 0 mN) were excluded (what was done with RP tissues) (Fig. 2c), contractions were again similar between tissues from HoLEP/ThuLEP and RP (HoLEP/ThuLEP 2.99 mN [2.66–3.32]; RP 3.33 mN [3.1–3.55]; MD 0.33 mN [-0.18 to 0.846]), but still substantially lower in TURP tissues. The percentage of single samples without reaction to highmolar KCl amounted to 60% in TURP tissues and 14% in tissues from laser enucleation.

### EFS-induced contractions

**Fig. 3:**
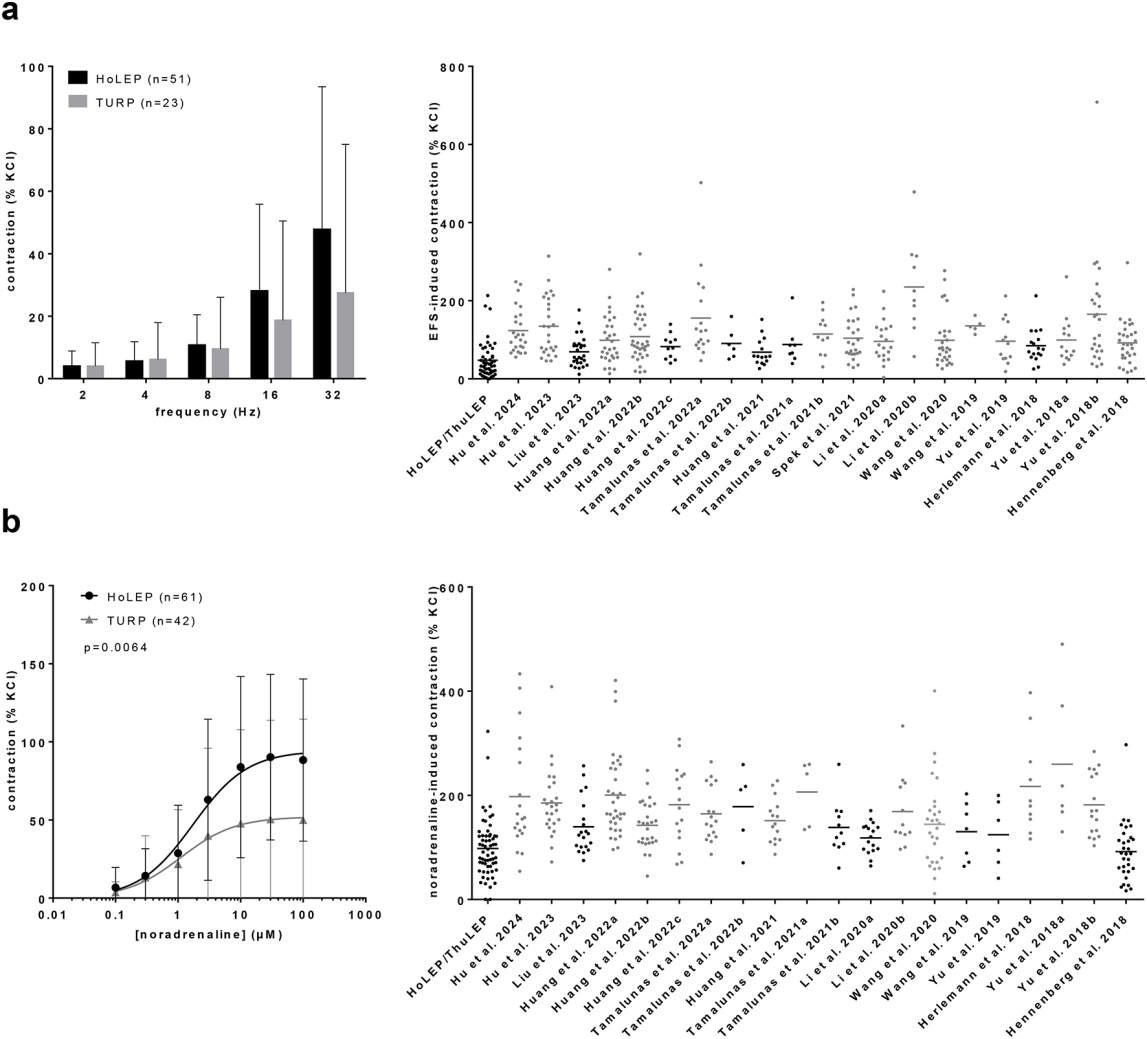
EFS- and noradrenaline-induced contractions in tissues from surgery for BPH, and from radical prostatectomy for PCa. Frequency response curves for EFS (**a**) and concentration response curves for noradrenaline (**b**) were constructed in tissues from laser enucleation (HoLEP, ThuLEP) and TURP. E_max_ values were calculated by curve fitting, for EFS (**a**) and noradrenaline (**b**). E_max_ for RP tissues from are from control groups in our previous studies, published from 2018 to 2023. Accordingly, contractions with RP tissues were induced in the presence of different amounts of solvent (mostly DMSO), used in controls for drugs examined in these previous studies, whereas contractions with tissues from laser enucleation and TURP were induced without solvent. Data in frequency and concentration response curves are means with standard deviation (SD). Frequency and concentration response curves were compared by two-way ANOVA. E_max_ values from RP tissues were compared by one-way ANOVA with Dunn’s test to E_max_ values from laser-enucleated tissues (grey data sets: *p* < 0.05 vs. HoLEP/ThuLEP; see supplementary table 1 for details).

EFS induced frequency-dependent contractions in laser-enucleated tissues (n = 51 patients) (Fig. 3a). E_max_ values were calculated by curve fitting and compared to E_max_ values from control groups (i. e., preincubated with solvents, but without drugs) in our previous studies with RP tissues (2018–2023). E_max_ values from HoLEP/ThuLEP tissues were not statistically different to E_max_ values for EFS-induced contractions of RP tissues in 6 from 21 of these previous studies (Fig. 3a). In 15 from 21 of studies with RP tissues, E_max_ values for EFS-induced contractions were higher compared to E_max_ values in tissues from HoLEP/ThuLEP (Fig. 3a). With an average E_max_ of 47% [35–60] of KCl-induced contractions, contractions of HoLEP/ThuLEP tissues ranged lower than E_max_ values in all studies with RP tissues, but with high overlap and with the closest E_max_ values in two studies with RP tissues ranging at 68% [49–88] and 69% [55–84] of KCl-induced contractions (Fig. 3a). The variability and range of maximum EFS-induced contractions in RP tissues was high across studies, with the highest E_max_ values mounting to 235% [150–320] and to 166% [107–224] of KCl-induced contractions (Fig. 3a). With an E_max_ of 27% [7–48], EFS-induced contractions of TURP tissues were obviously lower compared to laser-enucleated and to RP tissues (Fig. 3a).

### Noradrenaline-induced contractions

Noradrenaline induced concentration-dependent contractions in laser-enucleated tissues (n = 60 patients) (Fig. 3b). E_max_ values were calculated by curve fitting and compared to E_max_ values from control groups (i. e., preincubated with solvents, but without drugs) in our previous studies with RP tissues (2018–2023). E_max_ values in tissues from HoLEP/ThuLEP were similar (i. e., not statistically different) to E_max_ values for noradrenaline-induced contractions of RP tissues in 7 from 20 of these previous studies (Fig. 3b). In the 13 other from 20 of studies with RP tissues, E_max_ values for noradrenaline-induced contractions were higher compared to E_max_ values observed with tissues from HoLEP/ThuLEP (Fig. 3b). With an average E_max_ of 99.7% [85–114] of KCl-induced contractions, contractions of HoLEP/ThuLEP tissues ranged lower than E_max_ in 19 of the 20 studies with RP tissues, but with high overlap and with the closest E_max_ values ranging at 92% [71–112] and 118% [103–133] of KCl-induced contractions in two studies with RP tissues (Fig. 3b). The variability and range of maximum noradrenaline-induced contractions in RP tissues was high across studies, with the highest E_max_ values mounting to 260% [112–407] and to 217% [150–284] of KCl-induced contractions (Fig. 3b). With an E_max_ of 56% [34–79], noradrenaline-induced contractions of TURP tissues were obviously lower compared to laser-enucleated and to RP tissues (Fig. 3b).

### Grouping of EFS-induced contractions of HoLEP/ThuLEP tissues

**Fig. 4:**
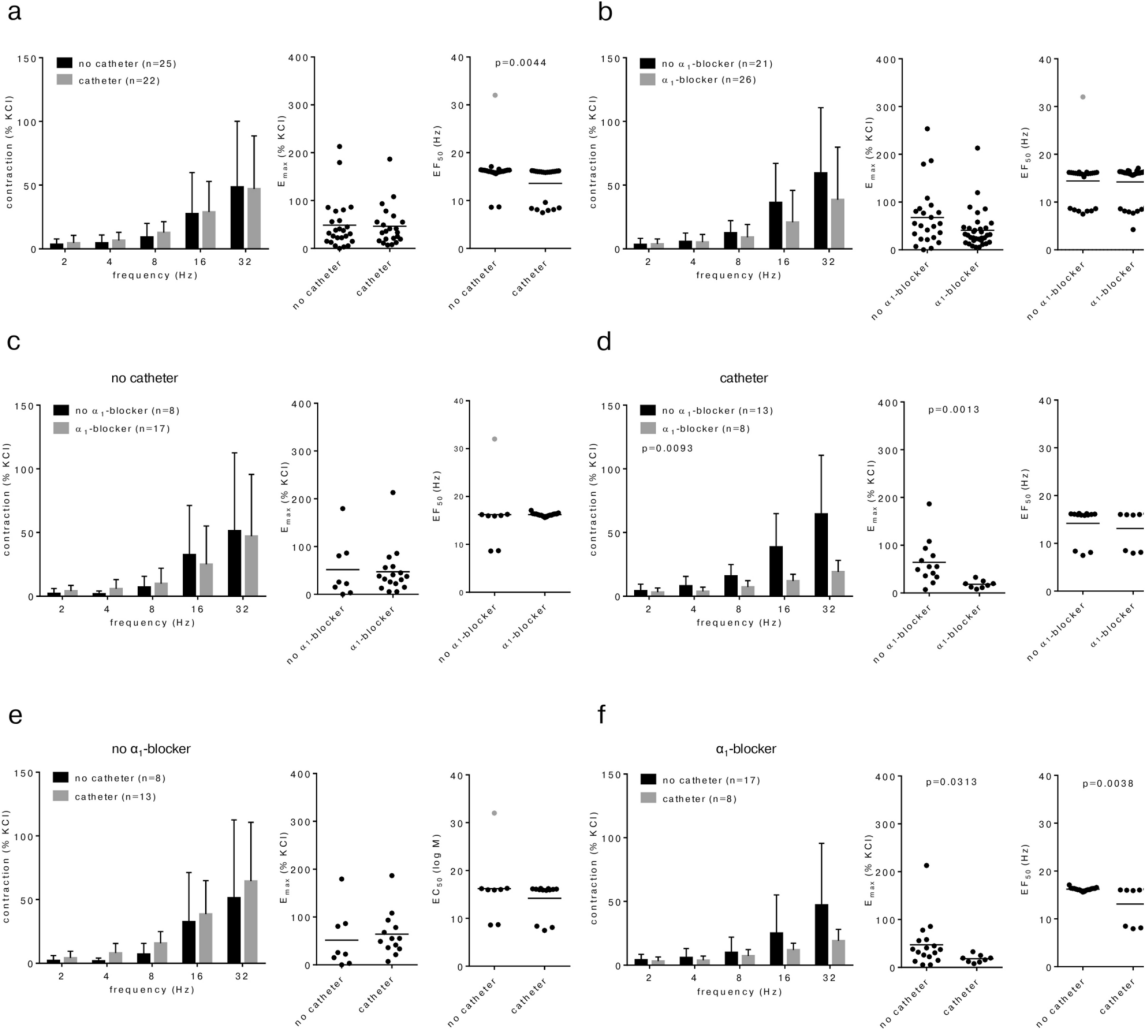
EFS-induced contractions in laser-enucleated tissues, grouped for preoperative catheterization and preoperative treatment with α_1_-blockers. Data from EFS-induced contractions were grouped as indicated, for preoperative catheterization of patients due to urinary retention (**a**), for preoperative treatment with α_1_-blockers (**b**), or both (**c-f**). Patients’ data regarding catheterization and medical treatment were available from 47 of a total of 51 patients participating in this series (25 without, 22 with catheter; 26 treated, 21 not treated with α_1_-blocker). Shown are means ± standard deviation (SD) in frequency response curves, and all single values for E_max_ and EF_50_ calculated by curve fitting (each value representing one prostate tissue, examined by single or multiple determinations) together with means (bars). Frequency response curves were compared by two-way ANOVA. E_max_ and EF_50_ values were compared by unpaired, two-tailed t test if data were normally distributed in both groups (i. e., EF_50_ values in (b) and (f)), and by unpaired, two-tailed Mann Whitney test if data were not normally distributed in at least one of both groups (all others). *P* values ≥ 0.05 are not shown. One EF_50_ value (labelled by grey color) could not be calculated by curve fitting, as the sample showed no contraction (corresponding to an E_max_ of 0% of KCl), but was set to the highest applied frequency, i.e. 32 Hz for illustration.

EFS-induced contractions were similar in tissues from patients without (n = 25) and with catheter (n = 22) (Fig. 4a). In patients receiving preoperative treatment with α_1_-blockers (n = 21), contractions were by trend (though, not significantly) lower compared to patients without treatment (n = 26) (E_max_ 68% [41–94] of KCl without α_1_-blocker, 41% [27–55] with α_1_-blocker, MD 27% [0–54]) (Fig. 4b). In patients without catheter, contractions were similar in tissues from patients with (n = 17) or without (n = 8) treatment with α_1_-blockers (Fig. 4c). However, in patients with catheter, contractions were substantially lower in patients with α_1_-blocker treatment (n = 8), compared to patients without α_1_-blocker treatment (n = 13) (Fig. 4d). Lower contractions in α_1_-blocker-treated patients were reflected by decreased E_max_ values (E_max_ 64% [36–92] of KCl without α_1_-blocker, 18% [11–25] with α_1_-blocker, MD 46% [11–81]) (Fig. 4d). Without α_1_-blocker treatment, contractions were similar in tissues from patients without (n = 8) and with (n = 13) catheter (Fig. 4e). In patients with α_1_-blocker treatment, contractions were lower in patients with catheter (n = 8), compared to tissues from patients without catheter (n = 17) (E_max_ 47% [22–72] of KCl without catheter, 18% [11–25] with catheter, MD 29% [-7 to 65]) (Fig. 4f).

### Grouping of noradrenaline-induced contractions in HoLEP/ThuLEP tissues

**Fig. 5:**
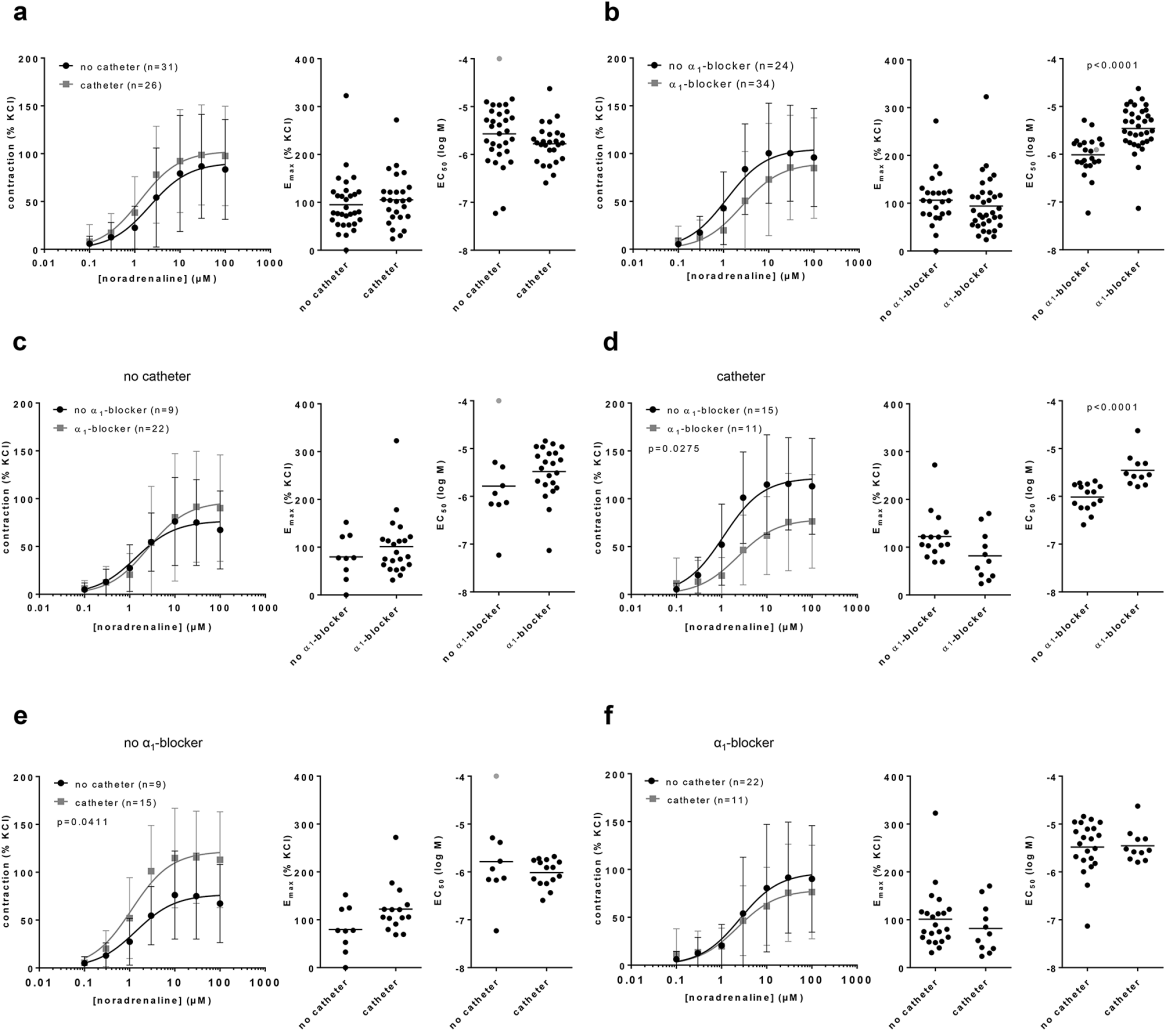
Noradrenaline-induced contractions in laser-enucleated tissues, grouped for preoperative catheterization and preoperative treatment with α_1_-blockers. Data from noradrenaline-induced contractions were grouped as indicated, for preoperative catheterization of patients due to urinary retention (**a**), for preoperative treatment with α_1_-blockers (**b**), or both (**c-f**). Patients’ data regarding catheterization were available from 57 patients (31 without, 26 with catheter), and regarding medical treatment from 59 patients (34 treated, 25 not treated with α_1_-blockers), from a total of 61 patients participating in this series. Shown are means ± standard deviation (SD) in concentration response curves, and all single values for E_max_ and EC_50_ calculated by curve fitting (each value representing one prostate tissue, examined by single or multiple determinations) together with means (bars). Concentration response curves were compared by two-way ANOVA. E_max_ and EC_50_ values were compared by unpaired, two-tailed t test if data were normally distributed in both groups (i. e., EC_50_ values in (a) and (e)), and by unpaired, two-tailed Mann Whitney test if data were not normally distributed in at least one of both groups (all others). *P* values ≥ 0.05 are not shown. One EC_50_ value (labelled by grey color) could not be calculated by curve fitting, as the sample showed no contraction (corresponding to an E_max_ of 0% of KCl), but was set to the highest noradrenaline concentration applied in these series, i.e. -4 for illustration.

Noradrenaline-induced contractions were similar in tissues from patients without (n = 31) and with catheter (n = 26) (Fig. 5a). In patients receiving preoperative treatment with α_1_-blockers (n = 34), contractions were by trend (though, not significantly) lower compared to patients without treatment (n = 24) (Fig. 5b). While E_max_ values for noradrenaline were similar between both groups, EC_50_ values for noradrenaline were increased in tissues from patients with α_1_-blocker pretreatment, compared to tissues from patients without pretreatment (pEC_50_ 6.01 [0.08–0.41] without α_1_-blocker, 5.44 [0.07–0.34] with α_1_-blocker, MD 0.57 [0.11–0.54]) (Fig. 5b). In patients without catheter, contractions were similar in tissues from patients with (n = 22) or without (n = 9) treatment with α_1_-blockers (Fig. 5c). While E_max_ values were similar between both groups, EC_50_ values for noradrenaline were increased by trend in tissues with α_1_-blocker treatment, compared to tissues from patients without treatment (pEC_50_ 5.79 [5.11–6.46] without α_1_-blocker, 5.48 [5.21–5.745] with α_1_-blocker, MD 0.31 [-0.4 to 1.02]) (Fig. 5c). In patients with catheter, contractions were substantially lower in patients with α_1_-blocker treatment (n = 11), compared to patients without α_1_-blocker treatment (n = 15) (Fig. 5d). Lower contractions in α_1_-blocker-treated patients were reflected by decreased (though, not significantly) E_max_ values (122% [94–151] of KCl without α_1_-blocker, 82% [48–116] with α_1_-blocker, MD 40% [2–82] of KCl), and by increased EC_50_ values for noradrenaline (pEC_50_ 6 [5.79–6.2] without α_1_-blocker, 5.45 [5.23–5.68] with α_1_-blocker, MD 0.54 [0.28–0.81]) (Fig. 5d). Without α_1_-blocker treatment, contractions were higher in tissues from patients with catheter (n = 15) compared to tissues from patients without catheter (n = 9) (Fig. 5e). Changes were reflected by E_max_ values, which were increased by trend (80% [43–116] of KCl without catheter, 122% [94–151] of KCl with catheter, MD 43% [-1 to 86] of KCl), while EC_50_ values for noradrenaline were similar between both groups (Fig. 5e). In patients with α_1_-blocker treatment, contractions were similar in tissues from patients without (n = 22) and with (n = 11) catheter (Fig. 5f).

### Ex vivo effects of α_1_-blockers on contractions in laser-enucleated tissues

**Fig. 6:**
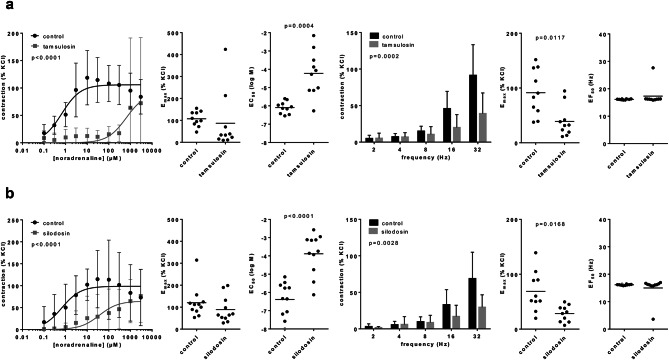
Ex vivo effects of α_1_-blockers on noradrenaline- and EFS-induced contractions of laser-enucleated tissues. Concentration response curves for noradrenaline and frequency response curves for EFS were constructed 30 min after application of solvent (controls) or 100 nM tamsulosin (**a**) or 100 nM silodosin (**b**) in tissues from laser enucleation (HoLEP, ThuLEP). E_max_, EC_50_ and EF_50_ values were calculated by curve fitting. Shown are means ± standard deviation (SD) in concentration and frequency response curves, and all single values for E_max_ and EC_50_ calculated by curve fitting (each value representing one prostate tissue, examined by single or multiple determinations) together with means (bars). Concentration and frequency response curves were compared by two-way ANOVA. E_max_ and EF_50_ values were compared by paired, two-tailed Wilcoxon matched-pairs signed rank test if data were not normally distributed in at least one of both groups (EF_50_ values, and E_max_ values for noradrenaline), and by paired, two-tailed t test if data were normally distributed in both groups (all others). *P* values ≥ 0.05 are not shown. One EC_50_ value marked in grey in (b) was calculated around -12, but has been included as -8 for better illustration (*P* value refers to data set including -12). Data are from n = 11 independent experiments for silodosin and EFS, and n = 10 independent experiments in each of the three other series, with the control and antagonist group in an independent experiment performed with tissue from the same patients, and with the number of experiments equating to the number of patients participating in a series. Experiments were performed with tissues from a total of 21 patients, not being included in other series of this study.

Effects of tamsulosin and silodosin, applied ex vivo in the organ bath, were examined in separate series with laser-enucleated tissues from 21 further patients. Both antagonists caused right shifts of concentration response curves for noradrenaline, increased EC_50_ values but unchanged E_max_ values for noradrenaline, and decreased E_max_ values for EFS (Fig. 6), without that further grouping was required. Tamsulosin increased the EC_50_ values for noradrenaline, from -6.09 [-6.32 to -5.86] in controls to -4.23 [-5.1 to -3.40] with tamsulosin (MD 1.87 [1.1–2.63] without reducing E_max_ values, and decreased the E_max_ for EFS-induced contractions from 91% [61–121] of KCl-induced contractions in controls, to 39% [19–59] with tamsulosin (MD -52% [-90 to -15]) without reducing EF_50_ values for EFS (Fig. 6a). Silodosin increased the EC_50_ values for noradrenaline, from -6.38 [-7.02 to -5.75] in controls to -3.89 [-4.64 to -3.13] with tamsulosin (MD 2.499 [1.87–3.13] without reducing E_max_ values, and decreased the E_max_ for EFS-induced contractions from 69% [43–95] of KCl-induced contractions in controls, to 28% [18–39] with tamsulosin (MD -40% [-71 to -9]) without reducing EF_50_ values for EFS (Fig. 6b).

## Discussion

To the best of our knowledge, this study is the first to address the smooth muscle contractility of tissues obtained by laser enucleation. Preclinical investigation of prostate smooth muscle contraction was crucial in the development of currently available drugs for treatment of voiding symptoms, including α_1_-blockers and the phosphodiesterase-5 inhibitor tadalafil. Meanwhile, limitations of these medications became evident^2^, initiating ongoing searches for novel targets and new candidate compounds, together with attempts to understand the reasons accounting for these limits. Consequently, contraction studies are of continuous interest. The findings of this study identify tissues from laser enucleation as a new model for investigation of prostate smooth muscle contraction, in patients with medication-refractory voiding symptoms in BPH.

Typically, patients selected for surgery for BPH are characterized by severe symptoms, while treatment with α_1_-blockers is recommended for patients with moderate to severe symptoms^3^. Ablative surgery is performed if complications of BPH are experienced, but also for adequate relief from lower urinary tract symptoms (LUTS) or postvoid urine in non-responders for medical treatment, or if medical treatment is refused but active treatment requested^3,4^. Thus, effective medical treatment is not available for these populations. Previous studies addressing prostate smooth contractions either used tissues from TURP or RP for PCa. TURP tissues cover the same or a similar patient group as laser-enucleated tissues. However, their use was often supposed to be limited by heat-induced traumatization, reducing their contractility in vitro. In our hands, TURP tissues were characterized by lower contractions compared to laser-enucleated tissues, and by an exceeding rate of complete non-responsiveness to contractile stimulation. While previous studies obtained results from TURP tissues, also with unquestioned relevance, information about exclusion and percentage of non-contractile tissues was rarely reported. In turn, tissues from RP for PCa, but without previous surgery for BPH were widely used in our previous studies. According to the prevalence of BPH in this age group^1^, BPH and mild symptoms are likely in these patients, but these tissues from RP do not specifically cover the context of severe or medication-refractory voiding symptoms. As contractions of laser-enucleated tissues approached ranges of contractions seen with RP tissues, without high rates of non-responsiveness due to traumatization by surgery, these tissues may provide a suitable model to study prostate smooth muscle contraction in patients with severe and medication-refractory LUTS. Finally, it allows to correlate in vitro findings with clinical data in the future, while BPH-specific data including international prostate symptom score (IPSS), Q_max_ or postvoid residual urine volume are not routinely assessed in patients undergoing RP for PCa.

The reasons accounting for the divergent impacts of preoperative α_1_-blocker treatment, seen between patients with and without catheterization for urinary retention are still elusive at this stage. Pretreatment with α_1_-blockers resulted in reduced contractions by EFS and noradrenaline in tissues from catheterized patients, but not in tissues from patients without catheterization. Certainly, this difference reflects substantial, yet unknown heterogeneity between both populations, even though both groups receive surgery for BPH. Reasons may include 1) different tissue responsiveness to α_1_-blockers, 2) different tissue conditions affecting the drug availability in tissues ex vivo, or 3) differences affecting drug metabolism and bioavailability in vivo, and 4) further reasons and combinations. A different tissue responsiveness to α_1_-blockers could be imparted by divergent receptor expression, or by unknown differences in receptor regulation^29^. Considering that ex vivo application of α_1_-blockers caused full effects, without grouping of patients, such differences in tissue responsiveness may be regarded as unlikely to impart the difference seen between catheterized and uncatheterized patients. Nevertheless, this cannot fully be excluded, as the experimental design differed for preoperative and ex vivo application of α_1_-blockers, including comparison of different patients in one series, but a comparison of paired samples from the same patients in the other series.

Divergent tissue conditions may provide plausible explanations for the different impact of preoperative α_1_-blocker treatment in both groups. BPH may include stromal, glandular and mixed hyperplasia, but their specific contributions to symptoms and drug responsiveness are not understood and have been poorly documented in preclinical and clinical studies^30^. Similar, prostatic fibrosis is a just recently emerging topic, and may include progressive deposition of extracellular matrix (ECM) compounds^31–34^. Drug penetration into tissues may reduce with increasing ECM content, depending on but also independently from vascularization in these tissue parts. This may reduce drug availability in vivo and thus, affect ex vivo contractility. Other factors related to tissue conditions may account as well. Different stromal-epithelial contents may determine the washout of α_1_-blockers during ex vivo procedures. Washout may be less effective in stromal compartments, and thus in tissues with high stromal content, compared to spongy tissues with high glandular content. With all due caution and if proving true, this may point to impaired washout of α_1_-blockers during ex vivo handling and thus, to predominant stromal hyperplasia in patients with catheterization (i.e. with urinary retention), and to predominant glandular hyperplasia in patients without. High individual variation in tissue consistency becomes obvious during routine work with TURP and laser ablation, ranging from soft to stiff tissues. In the run-up of the study, it was speculated that α_1_-blockers in the tissues, resulting from medication for voiding symptoms, will be washed out during surgery, intravesical maceration, transport and storage in custodiol solution, and finally by Krebs-Hensleit solution in the organ bath. Together, different tissue composition by cellular and non-cellular constituents, but also the phenotype of BPH may affect drug penetration and their removal in tissues, and may decide about clinical characteristics including urinary retention. Notably, the observable ex vivo effects from preoperative α_1_-blocker treatment seen in tissues from catheterized patients prove that the treatment affects prostate smooth muscle contractility, but without improving symptoms or complications, so these patients are evidentially medication-refractory.

Results from application of α_1_-blockers ex vivo demonstrated that tissues from laser-enucleation are suitable for investigation of drug effects in the context of BPH. Application of α_1_-blockers in the organ bath still resulted in full potential effects, including increases in EC_50_ values for α_1_-adrenergic agonists and recovery at high agonist concentrations. Considering that these patients needed surgery, despite treatment with α_1_-blockers and despite the effects of α_1_-blockers ex vivo, this raises the question, of whether inhibition of α_1_-adrenergic contractions, or smooth muscle contractions at all is a suitable strategy in these patients. For α_1_-adrenergic contractions, this is not the case. In addition to α_1_-adrenoceptors, prostate smooth muscle contraction can be induced by endothelins and thromboxane, to the maximum possible force. These non-adrenergic contractions were proposed to maintain full smooth muscle tone and thus, symptoms in medication-refractory LUTS^2,35^. Consequently, it appears possible that full inhibition of adrenergic contractions is insufficient for clinical effects, even if smooth muscle tone contributes to symptoms. On the other hand, it appears possible as well, that symptom severity is unrelated to smooth muscle contraction, even though this concept has been claimed for decades, but in view that a causative role of bladder outlet obstruction and symptoms has been challenged^2^.

While technical traumatization had lower effects on contractility in laser-enucleated tissues than in TURP tissues, influences of chopping, maceration and handling can not be fully excluded. TURP has been the gold standard in surgery for BPH for decades^3,36^. Laser-enucleation still has a niche existence in comprehensive healthcare^36,37^, but may gain in popularity, reaching a share of 20% in surgeries for BPH performed in France in 2018, and may increasingly replace TURP or emerged as the preferred option in centers with corresponding expertise^38–40^. HoLEP and ThuLEP equally relieve voiding symptoms, with high efficacy and safety^41^. Heat generation in HoLEP and ThuLEP occurs by absorption of laser radiation by the prostate tissue, leading to heating and vaporization of water within the tissue. Comprehensive data allowing direct comparisons to TURP are limited for laser-enucleation, but heat development and propagation within the prostate and in the suprapubic region appear limited with HoLEP and ThuLEP^42–44^. Ex vivo, increases in temperatures across the instrument shaft and within the enucleation cavity remained below 5 K at any examined irrigation (apart from 0 ml/min), which is insufficient to cause tissue damage^43^. Injurious temperatures are probably not attained during clinical application^45,46^. The depth of necrotic zones and tissue coagulation typically varies between studies and conditions for TURP^47^, and probably for laser enucleation as well, where it may range from 0.1 to 4 mm^43,48^. Together, higher contractions of laser-enucleated tissues, compared to tissues from TURP may reflect a lower degree of heat-induced traumatization during surgery.

While the observed differences between subgroups are of potential clinical relevance, the study is associated with limitations. The differences between patients with and without catheterization were surprising and obvious, but their investigation was not the primary aim. Thus, the initial, primary endpoint was the contractility in laser-enucleated tissues, in comparison to widely-used human tissue models. Unlike previous studies in which contractions were measured with solvent (as controls for test compounds), contractions were measured without further intervention in the current study. As another limitation, the study design does not take into account that male LUTS include storage symptoms caused by the urinary bladder, in addition to voiding symptoms attributed to BPH, or that conditions similar to voiding symptoms can be caused by an underactive bladder, instead of obstruction. A non-negligible number of patients with voiding symptoms (approximately 50%) also show detrusor overactivity and associated storage symptoms including urgency or frequency^49,50^. Clinical conditions and etiology of storage symptoms are highly variable, and may include or affect cholinergic voiding contractions of the detrusor, and non-cholinergic detrusor microcontractions initiating the micturition reflex^51^. Storage symptoms in male mixed LUTS may develop secondary to obstruction or independently from it, and persist after desobstructive surgery. In no case, however, detrusor contractions are induced by α_1_-adrenoceptors, explaining why storage symptoms are resistant to α_1_-blockers^51^. Consequently, symptom resistance to BPH-specific drugs may have been attributed to a certain, though unknown part of the study population, in particular in participants with high initial storage symptom scores, or with an underactive bladder. In fact, definite separation of voiding symptoms caused by obstruction from symptoms resulting from underactive or overactive bladder requires diagnosis by invasive urodynamics, or specific assessment of storage symptom scores^3,52^. In real world settings, decisions for desobstructive surgery are commonly based on routine care for male LUTS, not including diagnosis by invasive urodynamics^49^. It has been estimated, that 18–28% of patients undergoing prostate surgery for LUTS have no obstruction^53^. Symptoms in these patients may be predominantly attributed to bladder malfunction, and the surgery may be potentially unnecessary^49,53^. Thus, studies using tissues from desobstructive surgery addressing truly medication-refractory voiding symptoms should integrate data from diagnosis for storage symptoms.

## Conclusions

Smooth muscle contractions are intact in laser-enucleated prostate tissues, allowing investigation of prostate smooth muscle contraction in the context of medication-refractory voiding symptoms. Other than in TURP tissues, intrasurgical tissue traumatization does not limit investigation of contractility. Impacts of preoperative α_1_-blocker treatment divergently affected ex vivo contractility in tissues from patients with and without catheterization, reflecting fundamental differences in tissue conditions between patients with and without urinary retention. Heterogeneity in BPH differentially affects the risk for urinary retention in both subgroups, by unknown factors and despite shared surgery by laser-enucleation.

## Supplementary Information

Supplementary Information.

## Data Availability

All data that support the findings of this study are included in this published article. Raw data are available from the corresponding author upon reasonable request.
